# The war next-door—A pilot study on Romanian adolescents’ psychological reactions to potentially traumatic experiences generated by the Russian invasion of Ukraine

**DOI:** 10.3389/fpsyg.2022.1051152

**Published:** 2022-12-05

**Authors:** Alexandra Maftei, Oana Dănilă, Cornelia Măirean

**Affiliations:** Faculty of Psychology and Education Sciences, Alexandru Ioan Cuza University, Iași, Romania

**Keywords:** war, Ukraine, adolescents, emotional outcomes, anxiety, resilience

## Abstract

**Introduction:**

Romania shares the longest UE border with Ukraine, and since the Russian invasion of Ukraine began, many have been involved in helping the refugees. Consequently, children and adolescents might be directly and indirectly exposed to war-related trauma. In the present exploratory research, we investigated Romanian adolescents’ potential risk and protective factors related to the psychological outcomes of war exposure. Our cross-sectional study was conducted shortly after February 24th (i.e., the first invasion day).

**Methods:**

The sample included 90 Romanian adolescents aged 11 to 15 (M = 12.90, SD = 1.17), residents in Iași, Romania (i.e., 205,7 km from the Ukrainian border). Participants completed self-reported measures of peritraumatic dissociative experiences, knowledge about the conflict in Ukraine, personal, school, and family implications in volunteering/helping behavior, discussions about the conflict, threat perception (self and perceived parental threat), anxiety, social media engagement, resilience, and moral elevation.

**Results:**

The main findings suggested that participants involved in helping behaviors toward Ukrainian refugees present higher peritraumatic dissociative experiences, anxiety symptoms, and higher moral elevation than boys and participants not involved in these behaviors. Moreover, anxiety symptoms were positively associated with threat perception, peritraumatic dissociation, and social media engagement and negatively related to resilience.

**Discussions:**

Finally, we discuss the implications of our findings concerning their practical utility in managing peritraumatic exposure to war by using interventions designed to increase adolescents’ resilience during difficult times.

## Introduction

February 24th, 2022 marked the beginning of the Russian invasion of Ukraine. Initial findings suggested that this war caused deep physical and psychological trauma to its citizens (Gonçalves Júnior et al., 2022), along with severe damage to the civilian infrastructure, the country’s economy, the environment, and finally, to its freedom and democratic values (de Matos Brasil et al., 2022; Gonçalves Júnior et al., 2022; Pereira et al., 2022). Romanians have rapidly mobilized to help Ukrainian refugees since the war started, through various civic actions, in addition to the subsequent governmental measures (Havana, 2022). The large-scale Romanians’ mobilization included public protests against the Russian invasion, fundraising actions and calls, the creation of Facebook groups for the coordination of the aid effort, convoys transporting supplies to border crossings, and numerous offers of transportation and shelter (Anghel and Trandafoiu, 2022). According to Jawaid et al. (2022), those who are now participating in humanitarian rescue operations – such as the Romanians - face the risk of secondary trauma due to their constant exposure to others’ trauma. Similar previous findings were reported by studies exploring the posttraumatic reactions among rescue personnel (e.g., Hăisan et al., 2022).

The consequences of war trauma have been studied in various studies, highlighting the long-term impact on the victims and their families (e.g., Edward et al., 2020). Previous studies suggested that those who witness violence during the early stages of life (childhood and adolescence included) are at an increased risk of developing posttraumatic stress disorder (PTSD) should they experience another traumatic event while in adulthood (Breslau et al., 1999). The present research focused on the potential emotional consequences and the related risk and resilience factors concerning Romanian adolescents in the context of the Russian invasion of Ukraine. More specifically, we were interested in exploring Romanian adolescents’ reactions to the war at their country’s border.

Previous research also highlighted a significant link between peritraumatic dissociation (i.e., one of the most critical acute reactions to a traumatic event; Măirean, 2016) and PTSD among people repeatedly exposed to an armed conflict, such as the war in Ukraine (Duagani Masika et al., 2019). Similarly, studies on children and adolescents suggested that war exposure and mortality salience are significantly linked to elevated levels of depression and anxiety (Karam et al., 2014). In addition, in the case of war-related trauma exposure, peritraumatic dissociative experiences are common effects among youth (Peltonen et al., 2017). Depersonalization, a lack of subjective feelings, out-of-body experiences, and altered pain sensations are all symptoms of peritraumatic dissociation, causing a distorted sense of time and place (Schauer and Elbert, 2010; Peltonen et al., 2017).

The link between peritraumatic dissociative experiences and anxiety has been extensively studied in various contexts, from parental stress related to their children’s health (e.g., Bronner et al., 2009), natural disasters (Duncan et al., 2013) to the clinicians fighting the COVID-19 pandemic (Azoulay et al., 2020). Given the missing data regarding the war in Ukraine, we aimed to fill this gap by exploring the link between peritraumatic dissociative experiences and anxiety among Romanian teenagers exposed to this conflict.

### Threat perception: The role of social media engagement

Collective threats primarily refer to military, economic, and political threats, while individual threats might refer to the perceived potential adverse repercussions for one’s physical safety, personal health, or wealth (Rousseau and Garcia-Retamero, 2007). Regardless of age or developmental status, we perceive and react to threatening situations and detect *modern threats*, such as fire weapons (LoBue et al., 2010). When discussing war-related threat perception (i.e., war as a direct traumatic factor), previous studies suggested that adolescents threatened by military conflicts are generally reporting lower levels of well-being (Ronen and Seeman, 2007), decreased life satisfaction (Shamai and Kimhi, 2006), and higher levels of stress (Shamai and Kimhi, 2006). Furthermore, higher levels of perceived parental stress (due to increased threat perception) in military conflict-related contexts seem to be significantly associated with higher stress levels in adolescents (Shamai and Kimhi, 2007). However, gender appears to moderate these relationships, as well as the symptoms of PTSD due to military conflict exposure (with females generally reporting higher means; Armour et al., 2011).

As growing research suggests, social media engagement is essential in modeling threat perception in various contexts. For example, more information related to the threat (e.g., COVID-19) seem to be related to a higher perceived threat (e.g., Yang et al., 2020). The Russian-Ukrainian conflict has been called the world’s “First TikTok War” and “The Most Online War of All Time Until the Next One” (Chayka, 2022). Both parties are using social media to share information related to the conflict, and photos of the atrocities committed in the assaulted Ukrainian cities have millions of views on various social media platforms, from Facebook to Twitter and Tik Tok (Chayka, 2022). Investigations have already revealed that TikTok is also feeding war-related disinformation and fake content to new users, even if they do not search for Ukraine-related content (Cadier et al., 2022).

As Jawaid et al. (2022) suggested, billions of people worldwide are despairingly watching this battle theater (through social media and news, we would add) and have expressed emotions of powerlessness regarding the situation. Furthermore, this prolonged exposure to helplessness is a major risk factor for depression and other psychological disturbances, even more at present, after two pandemic years (Plomecka et al., 2021; Jawaid et al., 2022).

### War exposure and resilience

Resilience can be described as a process leading to positive adaptation in the face of significant adversity (Kim-Cohen, 2007) and is vital for survival and evolution (Fayyad et al., 2017). Previous research on children and adolescents exposed to trauma revealed several factors underlying this process. For example, a positive image of self, perceived family and social support, and positive family relations seem to act as protective factors against the development of PTSD and other adverse psychological symptoms (Brajša-Žganec, 2005; Tol et al., 2013).

As Fayyad et al. (2017) suggested, war-exposed teenagers seem more resilient when they use problem-solving skills, participate in leisure activities, have parents who spend time with them and provide academic support. Also, these associations seem to be stronger for males (Fayyad et al., 2017). Furthermore, previous studies suggested that teacher-student communication focused on emotional support during war exposure might contribute to adolescents’ resilience (Ophir et al., 2016). Similarly, classroom-based intervention programs delivered by teachers can effectively reduce negative reactions and strengthen student resilience in the face of traumatic events, such as war (e.g., Werner, 2012).

### Trauma and moral elevation

Finally, moral elevation describes a positive emotional state triggered by observing an act that one would consider highly virtuous, such as acts of charity, compassion, or perseverance (McGuire and Mignogna, 2021). These witnessed experiences might inspire and uplift the observer (Algoe and Haidt, 2009), being generally followed by the aspirations to imitate the observed virtue and become a better person (Oliver et al., 2012).

Numerous studies have suggested that exposure to elevating stimuli increases prosocial behavior (Van de Vyver and Abrams, 2015), positive affect, social interaction (Erickson et al., 2017), resilience and well-being (Caska and Renshaw, 2013), while lowering anxiety and depression (Erickson and Abelson, 2012). Furthermore, experimental investigations suggested that people with PTSD who were subsequently exposed to moral elevation stimuli might report more positive cognitions about others and the world, feel inspired, and engage in more compassionate goals (McGuire and Mignogna, 2021). Also, individuals exposed to trauma (i.e., mass shootings) who endorsed elevation reported increased compassion toward others and higher posttraumatic growth (Tingey et al., 2017). Thus, elevation is associated with resilience, improved psychological health, and social involvement, even in the case of trauma exposure, by providing the opportunity to directly target the negative effects of PTSD through cognitive, emotional, and behavioral experiences linked to trauma recovery (McGuire et al., 2019).

In the present research, we considered that witnessing the virtuous acts of the Romanians at the border, especially those near the participants in our sample (who were all adolescents from a town very close to the Ukrainian border), reading or discussing them through social media, peer or school groups, might be the moral elevation trigger proposed in McGuire et al. (2019) theoretical framework, further leading to the activation of positive-valence systems and, finally, the strong desire to imitate the witnessed behavior.

### The present study

The present study aimed to investigate Romanian adolescents’ emotional outcomes concerning the Ukrainian conflict (i.e., peritraumatic dissociative experiences, anxiety symptoms, and threat perception). Given the exploratory nature of our research and the novelty of the war-related context, our approach explored the potential connections between these variables of interest, to shape a comprehensive view of these possible connections. In addition, we explored the potential protective and risk factors in this regard, i.e., adolescents’ resilience, personal, family, and school implications in volunteering/helping behavior toward the Ukrainian refugees, social media engagement, information sources, and discussions about the conflict, familiarity/closeness with Ukrainians, and demographic factors. Finally, another aim of this research was to assess whether others’ implications in volunteering/helping behavior toward the Ukrainian refugees might lead to moral elevation among adolescents.

Given the previous findings concerning our variables of interest, our exploratory assumptions were the following:

*H1*: More frequent discussions about the conflict would be associated with higher resilience (through perceived social support; Ophir et al., 2016), especially in the case of male participants (Fayyad et al., 2017);*H2*: Female teenagers would report higher levels of peritraumatic dissociative experiences, threat perception, and anxiety than male participants (Armour et al., 2011); *H3*: Higher levels of perceived parental threat perception would be significantly associated with participants’ higher perceived threat and anxiety (Shamai and Kimhi, 2007); *H4*: Personal, family, or school (teachers’) implications in volunteering/helping behavior would generate significant differences regarding peritraumatic dissociative experiences and anxiety symptoms (McGuire et al., 2019); *H5*: Higher knowledge about the military situation in Ukraine would be associated with increased social media engagement, which would further be linked to increased threat perception, peritraumatic dissociative experiences, and anxiety (Jawaid et al., 2022).

## Materials and methods

### Participants

Our sample included 90 Romanian adolescents aged 11 to 15 (*M* = 12.90, *SD* = 1.17). The sample was relatively balanced regarding gender (male participants = 47.52.2%, and female participants = 43.47.8%). They were residents in Iași, Romania, a city in the northeastern part of Romania, close to the Ukrainian border (i.e., 205,7 km). They came from two-parent families in the urban area. Their participation was voluntary, following their parents’ consent.

### Procedures

We started collecting our data 3 weeks following the beginning of the war in Ukraine, following the ethical standards of the Helsinki Declaration. The Ethical Board from the university where the authors are affiliated approved the research. We collected our data in person, using the paper-pencil method, following parental approval. We informed the participants that participation was voluntary, there were no right and wrong answers, their responses would only be used for the present research. And they could withdraw from the study at any time, that the data they would provide would be anonymous, and that all information would remain confidential. The time needed to answer all the items was around 25 min.

## The present study

### Measures

We used the back-translation procedure (Hambleton and Li, 2005) for some scales (i.e., scales assessing peritraumatic dissociative experiences, anxiety, social media engagement, and resilience). The minimal differences between the original and back-translated versions were reconciled, resulting in the final versions of each instrument.

### Peritraumatic dissociative experiences

We used the scale developed by Marmar et al. (1997), i.e., the Peritraumatic Dissociative Experiences Questionnaire (PDEQ), to measure peritraumatic dissociation. The 10-item self-reported questionnaire uses a 5-point Likert scale, ranging from 1 = strongly disagree to 5 = strongly agree. Participants were asked to think about how they felt the past few days and choose the answer that suited them the most. Cronbach’s alpha for this scale was.89.

### Knowledge about the conflict in Ukraine

We asked participants how well they knew the military conflict situation in Ukraine (i.e., How well do you know the situation in Ukraine?). They answered on a 5-point Likert scale, ranging from 1 = not at all to 5 = very well. Next, we also asked them about the sources of the information related to the situation in Ukraine (open-end question).

### Personal and family implications in volunteering/helping behavior

We further asked the participants whether they or their family members were involved in helping behavior toward the Ukrainian refugees since the conflict started – Yes/No answers. Next, we also asked them about the types of helping behavior (e.g., helping with food and hygiene products/offering transportation/offering money/medical assistance).

### School implication—volunteering/helping behavior

We asked the participants whether the school they belonged to was implicated in volunteering/helping actions (Yes/No questions).

### Discussing the conflict

We asked the participants about the frequency of the talks about the Ukrainian conflict with family, teachers, friends, classmates, and other family members. We used a 5-point Likert scale (from 1 = never to 5 = very often, every day; see Appendix A). Higher scores indicate higher talk frequency.

### Threat perception (self)

We used an adapted version of the scale developed by Marciano et al. (2022) to assess the perceived threats related to the war in Ukraine. We used a 5-point Likert scale, ranging from 1 (not at all) to 5 (very much). The four items are detailed in Supplementary Appendix A. Higher scores indicated a higher perceived threat. Cronbach’s alpha for this scale was 0.71, and the inter-item correlation mean was.39.

### Threat perception (parents)

In the case of the perceived parental threat (i.e., how participants perceived their parents’ threat concerning the war in Ukraine), we used the same items for adolescents but adapted them to this parental context. Higher scores indicated a higher perceived parental threat. Cronbach’s alpha for this scale was 0.79, and the inter-item correlation mean was.49. Also, we asked adolescents to indicate which parent (or close relative) they report these answers about.

### Anxiety

We used the 41-item Screen for Child Anxiety Related Disorders – Child version (SCARED), developed by Birmaher et al. (1999), the Romanian version (Măirean et al., 2022). The answers were given on a Likert scale ranging from 0 (not true or hardly true) to 2 (very true or often true). We used the overall score in the present study. Cronbach’s alpha for the overall scale was 0.87.

### Social media engagement

We used an adapted version of the Social Media Engagement Scale for Adolescents (SMEQ-A; Ni et al., 2020), referring to the use of social media to check information regarding the situation in Ukraine. The 11-item scale measures participants’ responses on a Likert scale ranging from 1 t (not at all true) to 5 (very true). Higher scores indicated higher social media engagement to check for news related to the war. Cronbach’s alpha in the present study was 0.90.

### Resilience

We used the Adolescent Psychological Resilience Scale, developed by Bulut et al. (2013). The 29-item scale measures adolescents’ resilience using a 4-point Likert scale ranging from 1 (not exactly suitable for me) to 4 (entirely suitable for me). We used the overall score of the scale. Higher scores indicated higher resilience. Cronbach’s alpha for the overall scale was 0.81.

### Moral elevation

We constructed two items to measure moral elevation in the context of the Ukrainian conflict, using moral scenarios similar to the ones used in previous studies (e.g., Zheng et al., 2019; see the Appendix). The inter-item correlation mean between the two items was 0.40.

We used a demographic scale to assess participants’ gender and age. Also, in the first section of the questionnaire, we asked the participants what/which were the primary sources of information regarding the conflict in Ukraine and what volunteering/helping behavior they (or their families) were involved in.

## Results

### Preliminary steps

Before beginning our data analysis, we screened the data for potential errors. There were no missing responses or modifications discovered in our data. Next, we analyzed the normality of the variables (i.e., Skewness and Kurtosis values ranging from [−2; 2]; George and Mallery, 2010). In our case, the data was normally distributed. Table 1 presents the descriptive statistics for all the primary variables. Table 2 describes the primary sources of information regarding the conflict in Ukraine.

**Table 1:** Descriptive statistics for all the primary variables (*N* = 90).

	M	SD	Min	Max	Skewness	Kurtosis
1. Knowledge about the conflict	3.78	0.78	1	5	−0.45	0.79
2. Threat perception (self)	9.25	3.34	4	19	0.48	0.29
3. Threat perception (parents)	10.57	3.97	4	20	0.17	−0.57
4. Discussions about the conflict	14.82	5.51	5	25	0.13	−0.69
5. Resilience	88.15	11.47	53	109	−0.54	0.44
6. Peritraumatic dissociative experiences	27.51	10.36	10	50	0.14	−0.78
7. Social media engagement	32.36	10.87	11	54	−0.21	−0.80
8. Moral elevation	9.22	3.32	3	14	−0.06	−1.11
9. Anxiety	69.88	17.83	46	123	0.92	0.67

**Table 2:** Primary information sources regarding the conflict in Ukraine and volenteering behaviors.

Information source	N	%
Press - TV	40	44.44
Press (TV) and school	2	2.22
Press (TV) and other people	2	2.22
Press (TV) and the Internet	7	7.77
Press (TV) and parents	4	4.44
Youtube	1	1.11
The Internet	5	5.55
Social media (*unspecified)*	1	1.11
Press and Tik Tok	7	7.77
Tik Tok and Youtube	1	1.11
Tik Tok and the Internet	1	1.11
Tik Tok and news (press)	19	
**Helping behaviors (volunteering)**		
I was not involved in volunteering/helping behaviors (neither was my family).	69	76.7
I/ We offered free housing	1	1.1
I/ We offered food, subsistence products, and hygiene products	7	7.8
I/ We offered free housing, food, and hygiene products	1	1.1
I/ We offered food, hygiene products, and donated money to NGOs	2	2.2
I/ We provided medical assistance	1	1.1
I/ We donated money to NGOs or similar entities in order to help the refugees	1	1.1
I/ We offered food and hygiene products	8	8.9

### Associations between the main variables

Correlation analyses (see Table 3) suggested that the knowledge about the conflict in Ukraine was positively associated with the discussions about the war (*r* = 0.39, *p* < 0.001), peritraumatic dissociative experiences (*r* = 0.25, *p* = 0.01), and moral elevation (*r* = 0.22, *p* = 0.03). Participants’ threat perception was positively associated with the perceived parental threat (*r* = 0.56, *p* < 0.001), peritraumatic dissociative experiences (*r* = 0.25, *p* = 0.01), anxiety (*r* = 0.34, *p* = 0.001), and negatively related to resilience. More frequent discussions about the war were associated with higher peritraumatic dissociative experiences (*r* = 0.35, *p* = 0.001) and social media engagement (*r* = 0.44, *p* < 0.001). Also, participants’ resilience was negatively associated with their anxiety (*r* = −0.25, *p* = 0.01). Furthermore, the reported peritraumatic dissociative experiences were significantly related to social media engagement (*r* = 0.29, *p* = 0.006), moral elevation (*r* = 0.26, *p* = 0.01), and anxiety (*r* = 0.36, *p* < 0.001). Finally, age was significantly linked to the discussions about the war (*r* = 0.27, *p* = 0.00) and social media engagement (*r* = 0.22, *p* = 0.03).

**Table 3:** Associations between the primary variables (*N* = 90).

	1	2	3	4	5	6	7	8	9
1. Knowledge about the conflict	1								
2. Threat perception (self)	0.06	1							
3. Threat perception (parents)	0.11	0.56**	1						
4. Discussions about the conflict	0.39**	0.14	0.03	1					
5. Resilience	−0.14	−0.32*	−0.04	−0.00	1				
6. Peritraumatic Dissociative Experiences	0.25*	0.25*	0.11	0.35**	−0.20	1			
7. Social media engagement	0.34**	−0.03	0.18	0.44**	−0.04	0.29**	1		
8. Moral elevation	0.22*	0.20	0.13	0.19	0.05	0.26*	0.17	1	
9. Anxiety	0.15	0.34**	0.19	0.19	−0.25*	0.36**	0.24*	0.18	1
10. Age	0.01	−0.17	−0.11	0.27*	0.02	0.00	0.22*	−0.04	−0.14

*p < 0.05; **p ≤ 0.001.

### Gender differences

We further tested for the potential gender differences concerning the primary variables of our study. Results suggested that girls in our sample reported significantly higher perceived threat (*M* = 10.02) than boys (*M* = 8.55), *t*(88) = −2.12, *p* = 0.03. A significant difference (*t*(88) = −1.96, *p* = 0.05) was also observed concerning participants’ anxiety, with girls (*M* = 73.69) scoring significantly higher than boys (*M* = 66.40). Finally, girls reported higher moral elevation (*M* = 10.32) than boys (*M* = 8.21), *t*(88) = −3.16, *p* = 0.002.

### Differences by self or family implication in volunteering/helping behavior

We tested for the potential differences concerning the primary variables of our study depending on participants’ implication (or their family’s implication) in volunteering/helping behaviors toward the Ukrainian refugees. Results suggested that the only significant differences were related to the peritraumatic dissociative experiences and moral elevation. Participants involved in such actions (or their families’ implication; *N* = 21) reported higher peritraumatic dissociative experiences, *t*(88) = −2.01, *p* = 0.04, and higher moral elevation, *t*(88) = −2.25, *p* = 0.02.

### Differences by school’s implication in volunteering/helping behavior

Next, we tested for the potential differences concerning the primary variables of our study depending on the participants’ school’s implication in volunteering/ helping behaviors toward the Ukrainian refugees. Similar to the previous analyses (concerning personal or family implications), the results suggested significant differences related to the peritraumatic dissociative experiences and moral elevation. Participants who studied in schools that were involved in such actions (or their families’ implication; *N* = 18) reported higher peritraumatic dissociative experiences, *t*(88) = −3.75, *p* < 0.001, and higher moral elevation, *t*(88) = −3.63, *p* < 0.001. Significant differences (*p* = 0.05) were also found concerning parental threat perception, *t*(88) = −1.92, and anxiety, *t*(88) = −1.96; in both cases, the school’s involvement generated higher scores.

## Discussion

The present study aimed to investigate Romanian adolescents’ psychological outcomes after the Russian – Ukrainian conflict outbreak.

Our results suggested that girls present higher perceived threat, anxiety, and moral elevation than boys, sustaining our assumption. Previous studies documented that female participants are more vulnerable to traumatic reactions (Armour et al., 2011). However, in addition to these previous findings, the current results showed that girls are more vulnerable to negative outcomes (i.e., perceived threat, anxiety) but also present high moral elevation. Therefore, our results suggested that this adverse event generated intense negative reactions, but participants were still able to develop a positive emotional state triggered by witnessing a highly virtuous act of charity, compassion, or perseverance (McGuire and Mignogna, 2021).

Furthermore, our results showed that participants involved in volunteering/helping behaviors toward the Ukrainian refugees reported higher peritraumatic dissociative experiences (sustaining our assumption), and higher moral elevation compared with participants not involved in these types of behaviors. Participants that reported that their schools are implicated in volunteering behaviors reported more threat perception and anxiety than participants that did not report such behaviors. These results also confirmed the limited previous literature about the risk of secondary trauma among persons participating in humanitarian rescue operations (Jawaid et al., 2022) but also extended this literature by documenting the high probability of developing moral elevation, not only negative reactions.

We also assumed that more frequent discussions about the conflict would be associated with higher resilience through perceived social support. Our results suggested that more knowledge about Ukraine’s conflict relates to discussions about the war, more peritraumatic dissociative experiences, and more moral elevation. Threat perception was positively related to the perceived parental threat, peritraumatic dissociative experiences, and anxiety. Moreover, as expected, threat perception was negatively related to resilience. Furthermore, the reported peritraumatic dissociative experiences were significantly associated with social media engagement, moral elevation, and anxiety. Some of these relations (i.e., dissociation, anxiety, and resilience) sustain previous literature (e.g., Bronner et al., 2009) and extend it by focusing on a less studied sample, i.e., adolescents. Also, an interesting result is represented by the positive association between social media engagement and anxiety symptoms, partially sustaining our last assumption. Although social media may be a source of information, it could also increase the vulnerability to unwanted emotional states, like anxiety symptoms.

The results of the present study can be targeted in interventions designed to increase adolescents’ resilience during difficult times. Paying attention to distressing emotional states in the present could prevent unwanted future long-term consequences for mental health. Addressing and increasing the positive emotions generated by moral elevation could also be a way to improve mental health among persons indirectly exposed to traumatic life events. Moreover, parents could be encouraged to discuss with their children and monitor their media exposure to protect children and adolescents during these times.

When interpreting these results, several limitations should be considered. First, our limited sample size impedes us from generalizing the results to a larger population, but offer us a limited picture of how Romanian teenagers might perceive the current context generated by the Russian – Ukraine conflict and may be expanded in future research. Second, we cannot identify causal relations between variables, given the cross-sectional nature of our study. Thus, prospective longitudinal studies with more time waves would help us to assess how emotional reactions toward the current context evolve in time. Next, our measures were self-reported, increasing the chances of desirability. Finally, we used two constructed items for moral elevation and not a specific scale, an issue that further studies might address using experimental approaches.

In order to further explore the risk and protective factors for adolescents’ mental health of exposure to war trauma, future studies should consider how personal factors (e.g., trait resilience, moral elevation) interact with event-specific factors (e.g., discussion about the war, knowledge about the conflict) in predicting states of anxiety and dissociation. Furthermore, it might be possible that, during these rough times, adolescents exposed to acts of goodwill (such as helping the refugees) might strengthen their moral identity, which is crucial considering the high pressure of war-related unpredictability. However, these assumptions need further exploration in subsequent research. Future research could also consider the role of school (i.e., teachers, peers) and family support in shaping adolescents’ reactions to difficult situations.

## Conclusion

In conclusion, the current results suggested that girls presented a higher perceived threat, anxiety, and moral elevation than boys. Moreover, participants involved in helping behaviors toward Ukrainian refugees might be more vulnerable to unwanted outcomes, such as higher peritraumatic dissociative experiences, threat perception, and anxiety symptoms. The present study also showed that anxiety is positively related to the discussion about conflict, peritraumatic dissociation, and social media engagement, while the relation with resilience is negative. Future studies are needed to identify moderator variables and to explain additional mechanisms regarding the emotional outcomes of indirect exposure to armed conflicts.

## Data availability statement

The raw data supporting the conclusions of this article will be made available by the authors, without undue reservation.

## Ethics statement

The studies involving human participants were reviewed and approved by Faculty of Psychology and Educational Sciences, “Alexandru Ioan Cuza” University (Iasi, Romania). The patients/participants provided their written informed consent to participate in this study.

## Author contributions

All authors listed have made a substantial, direct, and intellectual contribution to the work and approved it for publication.

## Conflict of interest

The authors declare that the research was conducted in the absence of any commercial or financial relationships that could be construed as a potential conflict of interest.

## Publisher’s note

All claims expressed in this article are solely those of the authors and do not necessarily represent those of their affiliated organizations, or those of the publisher, the editors and the reviewers. Any product that may be evaluated in this article, or claim that may be made by its manufacturer, is not guaranteed or endorsed by the publisher.
