# Supplementary material for: The war next-door—A pilot study on Romanian adolescents’ psychological reactions to potentially traumatic experiences generated by the Russian invasion of Ukraine
Source: Front Psychol. 2022 Dec 5;13:1051152. doi: 10.3389/fpsyg.2022.1051152 (PMC9762354; doi:10.3389/fpsyg.2022.1051152)
Supplement: Supplementary file 1 [file Data_Sheet_1.docx]

**The war next-door.**

**A pilot study on Romanian adolescents' psychological reactions to potentially traumatic experiences generated by the Russian invasion of Ukraine**

**Appendix**

**Note 1.**

It is important to mention that we did not use the word *war* when asking specific questions related to the conflict in Ukraine. Our decision was motivated by our intent to avoid any biases related to participants' knowledge and representation of the war at the border and protect them from emotional challenges related to the word's meaning.

**Note 2. Variable: Discussing the conflict**

We asked the participants about the frequency of the talks about the Ukrainian conflict. We used five questions in this regard and a 5-point Likert scale (from 1 = never, to 5=very often, every day). The questions were:

(1) ***Family (parents):*** Have you been talking to your parents at home about the situation in Ukraine lately (since the outbreak of the conflict)?

(2) ***Teachers***: Have you been talking to your teachers at school about the situation in Ukraine lately (since the outbreak of the conflict)?

(3) ***Friends:*** Have you been talking to your friends about the situation in Ukraine lately (since the outbreak of the conflict)?

(4) ***Classmates:*** Have you been talking to your classmates at school about the situation in Ukraine lately (since the outbreak of the conflict)?

(5) ***Family*** (other than parents): Have you been talking to your family (not necessarily your parents) about the situation in Ukraine lately (since the outbreak of the conflict)?. Higher scores indicate higher talk frequency.

**Note 3. Variable: Threat perception (self)**

We used an adapted version of the scale developed by Marciano et al. (2022) to assess the perceived threats related to the war in Ukraine. We used a 5-point Likert scale, ranging from 1 (not at all) to 5 (very much). The four items are detailed were as follows:

1) How much do you feel threatened these days by the security risks posed by the current geopolitical situation?

(2) How much do you feel threatened these days by the health risks posed by the current geopolitical situation?

(3) How much do you feel threatened these days by the economic risks created by the current geopolitical situation?

(4) How much do you feel threatened these days by the political risks created by the current geopolitical situation?.

**Note 3.** **Variable: Moral elevation**

Participants received the following instructions: "*Two situations are described below. Please read each of the two items and indicate how true the situations described for you are in the context of the recent conflict in Ukraine, knowing that 1 = not at all true for me; 7 = very true for me*". The two items were (1) "*I saw people involved in helping refugees in Ukraine (for example, I offer accommodation or a hot meal), and it made me want to do similar things in this context",* and (2) "*I was emotionally impressed with the way people got involved in helping refugees in Ukraine, and it made me want to do similar things in this context ".*
